# A three-dimensional deep learning model for inter-site harmonization of structural MR images of the brain: Extensive validation with a multicenter dataset

**DOI:** 10.1016/j.heliyon.2023.e22647

**Published:** 2023-11-23

**Authors:** Vincent Roca, Grégory Kuchcinski, Jean-Pierre Pruvo, Dorian Manouvriez, Xavier Leclerc, Renaud Lopes

**Affiliations:** a Univ. Lille, CNRS, Inserm, CHU Lille, Institut Pasteur de Lille, US 41 - UAR 2014 - PLBS, F-59000 Lille, France; b Univ. Lille, Inserm, CHU Lille, U1172 - LilNCog - Lille Neurosciences & Cognition, F-59000 Lille, France; c CHU Lille, Department of Neuroradiology, F-59000 Lille, France

**Keywords:** Brain MRI, Harmonization, CycleGAN, Brain volumetry, Radiomics, Brain age

## Abstract

In multicenter MRI studies, pooling the imaging data can introduce site-related variabilities and can therefore bias the subsequent analyses. To harmonize the intensity distributions of brain MR images in a multicenter dataset, unsupervised deep learning methods can be employed. Here, we developed a model based on cycle-consistent adversarial networks for the harmonization of T1-weighted brain MR images. In contrast to previous works, it was designed to process three-dimensional whole-brain images in a stable manner while optimizing computation resources. Using six different MRI datasets for healthy adults (n=1525 in total) with different acquisition parameters, we tested the model in (i) three pairwise harmonizations with site effects of various sizes, (ii) an overall harmonization of the six datasets with different age distributions, and (iii) a traveling-subject dataset. Our results for intensity distributions, brain volumes, image quality metrics and radiomic features indicated that the MRI characteristics at the various sites had been effectively homogenized. Next, brain age prediction experiments and the observed correlation between the gray-matter volume and age showed that thanks to an appropriate training strategy and despite biological differences between the dataset populations, the model reinforced biological patterns. Furthermore, radiologic analyses of the harmonized images attested to the conservation of the radiologic information in the original images. The robustness of the harmonization model (as judged with various datasets and metrics) demonstrates its potential for application in retrospective multicenter studies.

## Graphical abstract

**Figure:**
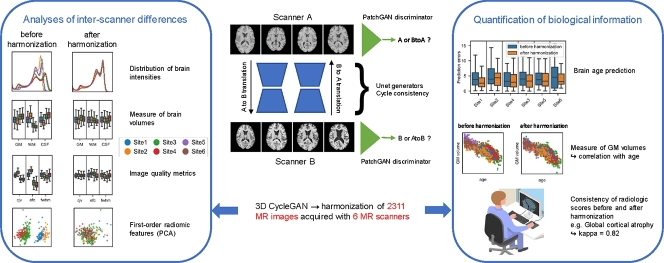

## 1 Introduction

Brain MRI is commonly used to diagnose various neurologic and psychiatric diseases. In order to detect subtle changes in neuroimaging features, researchers must increase the statistical power by studying large cohorts of patients in multicenter studies. However, the use of multicenter data introduces non-biological variations related to the MRI system's manufacturer, MRI field strength and site image quality [1], [2]. These limitations can mitigate the gains in statistical power. Despite efforts to standardize acquisitions, these unwanted variabilities can persist in the data [3], [4].

With a view to limiting the impact of site effects and retrospectively analyzing data from several studies, various harmonization methods have been developed based on feature- or image-level [5]. Statistical and machine learning approaches have been employed to effectively harmonize image-derived features such as brain volumes [6], [7], [8], radiomic features [9] and functional connectivity measures [10]. However, this type of approach relies on prior feature extraction and is not suited to the harmonization of whole brain MR images.

Image-level harmonization is mainly based on deep learning approaches and has shown encouraging results for structural MRI harmonization. Supervised learning using cohorts with “traveling subjects” (i.e. participants having been assessed at more than one center) have enabled to set up frameworks that do not require large datasets [11], [12] but are not applicable in many situations when such subjects are not available. Other types of harmonization methods have been designed for specific tasks [13], [14], [15] but require different training phases for each application and are limited to supervised predictions.

In the present study, we focus on the use of unsupervised deep learning frameworks for the harmonization of T1-weighted (T1w) brain MR images. CycleGAN [16] is certainly the most validated method. Although it requires retraining for each new site, it has shown encouraging results in harmonizing different imaging modalities and MR sequences [17], [18], [19], particularly when compared to statistical approaches [20]. Other deep learning models have been proposed to avoid retraining for every new site but didn't use site information [21], needed two different MR sequences as inputs for every subject [22] or were limited in terms of validation [23], [24].

Moreover, all these studies were based on 2D deep learning frameworks (or 2D models repeated on the three axes), which have the benefit of inducing fewer parameters to estimate than 3D frameworks. However, when it comes to 3D MR image harmonization, using 2D models inherently limits the quality of the generated images compared to 3D models. Palladino et al. [19] and Chen et al. [17] developed 3D solutions, but they were limited to low-volume patches. The resulting loss of spatial and contextual information was not justified or was dictated by material limitations [18], [19], [22].

Along with these methodological challenges, the evaluation of the quality of the harmonized results is also a key aspect. The main approach has been similarity metrics based on small traveling-subject datasets for which ground truth harmonization can be assumed [23], [25], [24], [18], [22]. Some studies also showed better prediction of biological information such as sex [18], [26], age [27], [26], disease [23], [25], [18] and brain tissue segmentation [17], [21], [19]. However, in most works the validations are not extensive enough to assess the robustness of the models to various applications. Furthermore, site effects have been reported for features extracted from T1w brain images: tissue volumes [28], radiomic features [29] and image quality metrics (IQMs) [30].

In this work, we propose a 3D CycleGAN model for the inter-site harmonization of T1w brain images that enables the processing of 3D images while preserving stability and optimizing computation resources. Using six datasets, we measured inter-site differences in intensity distributions, brain volumes, radiomic features and IQMs to evaluate our approach. We used age to quantify biological patterns through brain age prediction and the correlation between age and gray-matter (GM) volume, and we used specific scales to rate the conservation of radiologic patterns. We tested the model on cohorts with different age distributions and managed to harmonize them efficiently while avoiding overcorrection thanks to an appropriate training strategy. In addition, we validated the quality of the reconstructions with a traveling-subject dataset.

## 2 Materials and methods

### 2.1 Datasets

#### 2.1.1 Independent datasets

We obtained 3D T1w brain images from public data-sharing sources: IXI,^1^ OASIS-3 [31], NKI-RS [32] and NMorphCH [33] and then created six datasets of images acquired with six different machines (Table 1). All the participants were only present in a single dataset. As specified in the individual study protocols, all participants were healthy controls. Each participant had given his/her informed consent at the local study site, and each contribution was ethically approved.

**Table 1:** Characteristics of the participants and the scanner in each independent dataset.

Dataset name Study	Site1 IXI	Site2 IXI	Site3 OASIS-3	Site4 OASIS-3	Site5 NKI-RS	Site6 NMorphCH
**MR images, n**	309	176	984	453	248	141
**Participants, n**	309	176	405	345	246	44
**Age, years**⁎	50.75 ± 15.95	47.50 ± 16.63	69.37 ± 9.91	69.75 ± 8.69	30.00 ± 8.21	31.37 ± 8.42
**Males, %**	44	48	35	45	40	53

**Scanner model**	Philips Intera	Philips Intera	Siemens Magnetom TrioTim	Siemens BioGraph mMR PET-MR	Siemens Magnetom TrioTim	Siemens Magnetom TrioTim
**Field strength, Tesla**	1.5	3	3	3	3	3
**TR, ms**#	9.81	9.60	2400	2300 (423); 2400 (30)	1900 (184); 2600 (64)	2400
**TE, ms**#	4.60	4.60	3.16	2.95 (423); 2.13 (30)	2.52 (184); 3.02 (64)	3.16
**Resolution, mm**#	0.9 × 0.9 × 1.2	0.9 × 0.9 × 1.2	1.0 × 1.0 × 1.0	1.2 × 1.1 × 1.1 (423); 1.0 × 1.0 × 1.0 (30)	1.0 × 1.0 × 1.0	1.0 × 1.0 × 1.0
**Dimensions, voxels**#	256 × 256 × 150	256 × 256 × 150	176 × 256 × 256	176 × 240 × 256 (423); 176 × 256 × 256 (30)	176 × 256 × 256	176 × 256 × 256

^⁎^ Age is expressed as mean ± standard deviation.

^#^ The number of MR images with the corresponding parameter is indicated in brackets.

The selection of the MRI datasets is described in detail in the Suplementary Materials (section 1).

#### 2.1.2 Traveling-subject dataset

We selected 75 healthy subjects (CDR=0) from the OASIS-3 study who had been scanned at both Site3 and Site4 within three months to create a traveling-subject dataset for a supervised evaluation of site-related variabilities. These subjects were not included in the previously defined independent dataset (section 2.1.1).

### 2.2 MRI preprocessing

We first skull-stripped the T1w brain images (using volBrain software [34]) and corrected them for magnetic field inhomogeneity effects (using N4ITK algorithm [35]). Next, we linearly registered the MR images to 1 × 1 × 1 mm MNI space with FSL-FLIRT tool [36], [37] and scaled the intensities by setting the median within each brain to 500. We found median normalization to be less sensitive to outliers than maximum normalization, which is widely used when applying deep learning to MR images [23], [38], [39], [40], [41], [42]. To facilitate processing with CNNs, we padded and cropped the images to have dimensions 192 × 192 × 192 without removing any brain voxel.

### 2.3 Harmonization procedure

#### 2.3.1 Framework and architectures

The model developed is based on a CycleGAN configuration [16]. Given two image domains, it learns to translate an image from one domain to the other. The training needs a set of images from each domain but does not require ground truth (i.e. the two sets are not paired). With a multisite MRI dataset, one site is set as the reference and the images from the other sites are *transferred* towards the reference domain (MR images from the reference site are not modified).

**Figure 1:**
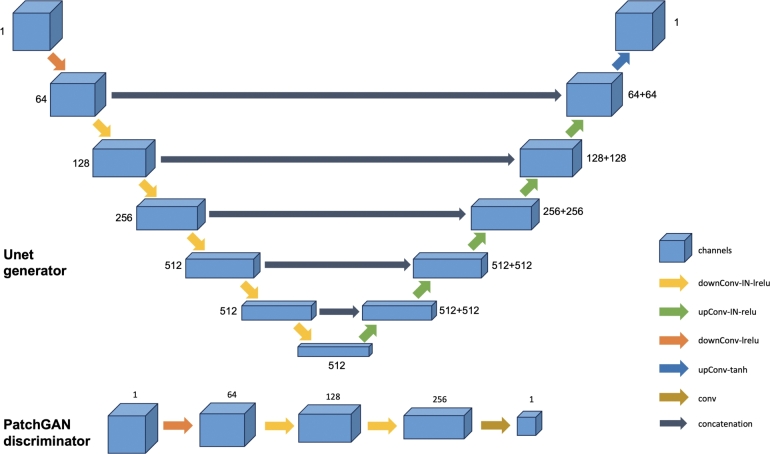
Network architectures of the proposed CycleGAN. Convolutions are 3D. The number next to each box indicates the number of channels. All the convolution kernels are 4 × 4 × 4 except in the last one of the discriminator which is 3 × 3 × 3. downConv: convolution with stride 2 × 2 × 2; upConv: transposed convolution with stride 2 × 2 × 2, IN: instance normalization; lrelu: leaky relu with a 0.2 slope.

We adapted the U-net generators and patchGAN discriminators described by Isola et al. [43] with 3D convolutions for processing whole 3D MR images (Fig. 1). The patchGAN discriminator had a receptive field of 38 × 38 × 38, which we found to be a good compromise between accessing contextual information and focusing on local imaging details. Inspired by Alami Mejjati et al. [44], we applied the original brain mask after every image generation to preserve brain structures and prevent background voxels from influencing the generator's training. The final activation function of the generator was linear for outputs above 0 (instead of tanh) to enable the generation of values above 1.

Both the generators and the discriminators took the entire 192 × 192 × 192 MR volumes as input.

#### 2.3.2 Model training

We implemented several strategies for increasing the training phase's stability and the model's robustness. Firstly, we pretrained a generator to replicate the input with all the datasets and an L1 loss; the generators of our CycleGAN were then initialized with the learned weights. Secondly, in order to make the early training stages less sensitive to variance, we also applied a linear decay from 200 to 100 for the hyperparameter weighting the cycle consistency constraint [45]. Thirdly, while we set the batch size to 1 for the generators, we stabilized the discriminators by training them with a history of 50 generated images [46] and at every step, each one was updated with 4 real images, 2 newly-generated images and 2 formerly-generated images.

In order to save GPU memory and speed-up the computations, we applied a mixed precision policy [47]. Inspired by Wu et al. [48], we replaced the standard deviation by the mean absolute deviation in instance normalization layers [49].

Further details of the training procedure are given in the Supplementary Materials (section 2).

### 2.4 Paired-site / multisite harmonizations

We used the six independent datasets (section 2.1.1) for the experiments described in this section.

#### 2.4.1 Configuration

In a first set of experiments, we harmonized three pairs of datasets: Site1 vs. Site2, Site3 vs. Site4, and Site5 vs. Site6. In each pair, the two sites had similar age distributions (statistical assessment in section 3 of the Supplementary Materials); this prevented confusion between site effects and biological variabilities during the training and evaluation steps. The three pairs were chosen to address various potential site effects: a difference in scanner field strength between Site1 and Site2, different MR scanners at Site3 vs. Site4, and a difference in acquisition parameters between Site5 and Site6 (Table 1).

We also performed an overall harmonization in which five sites were harmonized against a single reference site. Although Site1's images were acquired with a 1.5T scanner, we chose it as the reference site because it contributed a relatively large number of MR images and had a broad age range (from 20 to 86). Hence, we trained five models: Site1 vs. Site2, Site1 vs. Site3, ..., Site1 vs. Site6. To avoid correcting age effects because of differences in the datasets' age distributions (section 4 of the Supplementary Materials), we divided the MR images into several age ranges for each training session and assigned a probability of sampling for each age range; hence, at each step, the two training sets had the same probability of age distribution. To avoid undersampling the smallest populations, we developed an algorithm that, given the two training sets, gave us the probability of sampling in the age ranges. The algorithm is described in the Supplementary Materials (section 5), together with the sampling probabilities used for the various harmonizations vs. Site1. It should be noted that we did not adopt this sampling strategy for harmonization with Site2, since the two age distributions were similar.

#### 2.4.2 Quantitative evaluations

We measured various brain characteristics before and after harmonization to analyze site-related variabilities and biological information. To this end, we used FSL-FAST software [50] to segment GM, white-matter (WM) and cerebrospinal fluid (CSF).

##### 2.4.2.1 Site-related variabilities of MRI features

For a quantitative analysis of inter-site differences, we defined three groups of MRI features: tissue volumes, IQMs, and first-order radiomic features. The tissue volumes and IQMs are listed in Table 2 and were based on those described by Esteban et al. [30]. The 36 first-order radiomic features were extracted with the PyRadiomics tool [51] using the GM and WM masks. To limit the effect of redundant and noisy features, we applied standard scaling and principal component analysis (PCA) to project the radiomic features into 2D space and visualize potential site clusters.

**Table 2:** Tissue volumes and image quality metrics.

Tissue volumes	
icv_gm	GM, WM and CSF volumes normalized by the total intracranial volume.
icv_wm	
icv_csf	

**Image quality metrics**	
cjv	The coefficient of joint variation of the GM and WM intensities.
efc	The Shannon's entropy of voxel intensities.
snr_gm	The signal-to-noise ratio for GM, WM and CSF.
snr_wm	
snr_csf	
wm2max	The ratio between the WM's median intensity and the 95th percentile of the full-intensity distribution in the brain.
rpve_gm	An estimation of the residual partial volume effect for GM, WM and CSF.
rpve_wm	
rpve_csf	
fwhm	Full-width at half-maximum, an estimation of the blurriness of the image using AFNI *3dFWHMx*.


GM: gray-matter; WM: white-matter; CSF: cerebrospinal fluid.

Since these features can be influenced by age, some heterogeneities should not be removed by harmonization. In the multisite experiments, we therefore studied them on several specific age ranges (20-30, 50-60 and 60-70) separately. For each range, we included only sites with more than 20 participants.

##### 2.4.2.2 Brain age prediction

Brain age prediction consists in training a machine learning model to predict an individual's age from brain MRI data and has been widely investigated in recent years [52]. Deep learning methods can make accurate age estimations by processing images directly [53], [54]. However, the generalization of this type of model in medical imaging studies can be challenging [55]. In the present study, we implemented an age prediction model similar to that described by Cole et al. [53]; the goal was to evaluate the harmonization approach's ability to conserve or accentuate biological patterns.

For each of the three paired-site experiments, we selected the site with the largest number of MR images as the harmonization reference (i.e. Site1, Site3 and Site5), trained an age prediction model and evaluated the latter against the images from the other sites (i.e. Site2, Site4 and Site6) before and after harmonization. In order to measure baseline performances, we randomly split the three reference sites' MR images into training and test sets with age stratification; this resulted in 50, 117 and 30 test MR images for Site1, Site3 and Site5, respectively.

For the multisite experiment, we evaluated the model trained on MR images from Site1 (the reference set) against the other five datasets before and after harmonization. We also set up a brain age prediction model to assess the value of harmonization for a large multicenter training set. We randomly split our data into a training set of 1863 images and a nonoverlapping test set of 448 images, while conserving the proportion of images within each site. Next, we trained and evaluated two brain age prediction models: one without harmonization and the other with images harmonized against Site1.

To analyze the brain age predictions, we computed the mean absolute error (MAE) and the mean predicted age difference (MPAD, the average predicted age minus the real age). To account for regression towards the training mean [RTTM, 56] in the multisite experiment with the brain age model trained on Site1's MR images, we also computed the training mean deviation (TMD) by subtracting the mean age in the test set from that in the training set. The results were quoted in years.

Details of the training steps for the brain age prediction models are given in the Supplementary Materials (section 6).

##### 2.4.2.3 Correlation between age and gray-matter volume

One of the main characteristics of brain aging is a constant fall in GM volume throughout adulthood [57], [58], [59]. In order to assess the effect of harmonization on this aging pattern in the multisite experiment, we computed the linear correlation between GM volume and age.

##### 2.4.2.4 Radiologic assessments

In order to assess the conservation of radiologic patterns after harmonization, a subset of the T1w images were reviewed by a board-certified neuroradiologist (GK). The global cortical atrophy (GCA), medial temporal atrophy (MTA), enlarged perivascular spaces (EPSs) and ventricle size were assessed as these features are associated with normal aging and/or age-related disorders (such as Alzheimer's disease, small vessel disease or normal-pressure hydrocephalus). GCA was rated on a 4-point semi-quantitative scale, adapted from that described by Pasquier et al. [60] (0 = absent, 1 = mild, 2 = moderate, 3 = severe cortical atrophy) and MTA was rated on a 5-point semi-quantitative scale [61]. The EPSs were identified as small, linear, sharply delineated CSF intensities (or structures close to CSF intensities) measuring < 3 mm and that followed the course of perforating or medullary vessels [62]. The number of EPSs in the basal ganglia (EPS-BG, on the 1st slice above the anterior commissure) and the centrum semiovale (EPS-CS, on the 1st slice above the lateral ventricles) was rated as follows: 0 = no EPS, 1 = 1 to 9 EPS, 2 = 10 to 20 EPSs, 3 = 21 to 40 EPS, and 4 ≥ 40 EPS [63]. The Evans index was determined by the maximum transverse frontal horn ventricular width, perpendicular to the midsagittal line, on a 2D axial section parallel to the anterior commissure – posterior commissure plane, divided by the maximal transverse width of the intracranial cavity on the same plane [64].

For the paired-site experiments, 10 participants were randomly sampled from Site2, Site4 and Site5 and one image from each one was then rated before and after harmonization against Site1, Site3 and Site6, respectively. For the multisite experiment, 6 participants were randomly sampled from Site2, Site3, ..., Site6 and one image from each one was then rated before and after harmonization against Site1. The 120 rated MR images were shuffled and anonymized before the review. We quantified the consistency of the scores with harmonization by calculating the quadratic weighted kappa for the ordinal measures (i.e. GCA, MTA, EPS-BG, and EPS-CS) and the intraclass correlation coefficient (ICC) [65] for the Evans index. We interpreted kappa and the ICC in the following manner: poor below 0.40, fair between 0.40 and 0.59, good between 0.60 and 0.74, and excellent above 0.74 [66].

##### 2.4.2.5 Statistical inferences

We compared the sites with regard to the tissue volumes and IQMs by using two-tailed t-tests for the paired-site and a one-way analysis of variance (ANOVA) for the multisite experiments. For the age prediction results, we used two-tailed Wilcoxon signed-rank tests to compare the prediction errors before and after harmonization. We compared linear correlation coefficients using a two-tailed Steiger's test [67]. Using the Benjamini-Hochberg procedure, we corrected p-values for multiple comparisons (i) for each comparison of tissue volumes and IQMs, (ii) for the three brain age prediction comparisons in the paired-site experiments, and (iii) for the five brain age prediction comparisons in the multisite experiment with the model trained on Site1's MR images. To ensure independence between samples, we averaged the data for each participant in each test. The threshold for statistical significance was set to p < 0.05.

### 2.5 Harmonization on traveling subjects

We used our traveling subject dataset (section 2.1.2) to evaluate the ability of our model to transform images from one site to their equivalent in the other. We reused the harmonization models previously trained for the paired-site experiments (Site3 vs. Site4, section 2.4.1) to harmonize the 76 traveling subjects in both directions. We computed the structural similarity index measurement (SSIM) [68] for each image pair, with an intensity range fixed to 1000. Before computing SSIM, we removed the background slices.

We compared our 3D approach with a 2D CycleGAN adapted from Zhu et al. [16]. To exploit the three orientations, we trained three 2D models and generated the final output volumes with a 2.5D inference [23], [11]. For training, we used slices that contained above 1% of non-zero pixels after processing, following the approach of Bashyam et al. [27] and Cackowski et al. [23]. Further details of the 2D CycleGAN implemented are given in the Supplementary Materials (section 7). For each harmonization, we compared the SSIMs before and after harmonization with two-tailed Wilcoxon signed-rank tests and corrected the p-values using the Benjamini-Hochberg procedure.

## 3 Results

### 3.1 Paired-site experiments

#### 3.1.1 Image and histogram comparisons

**Figure 2:**
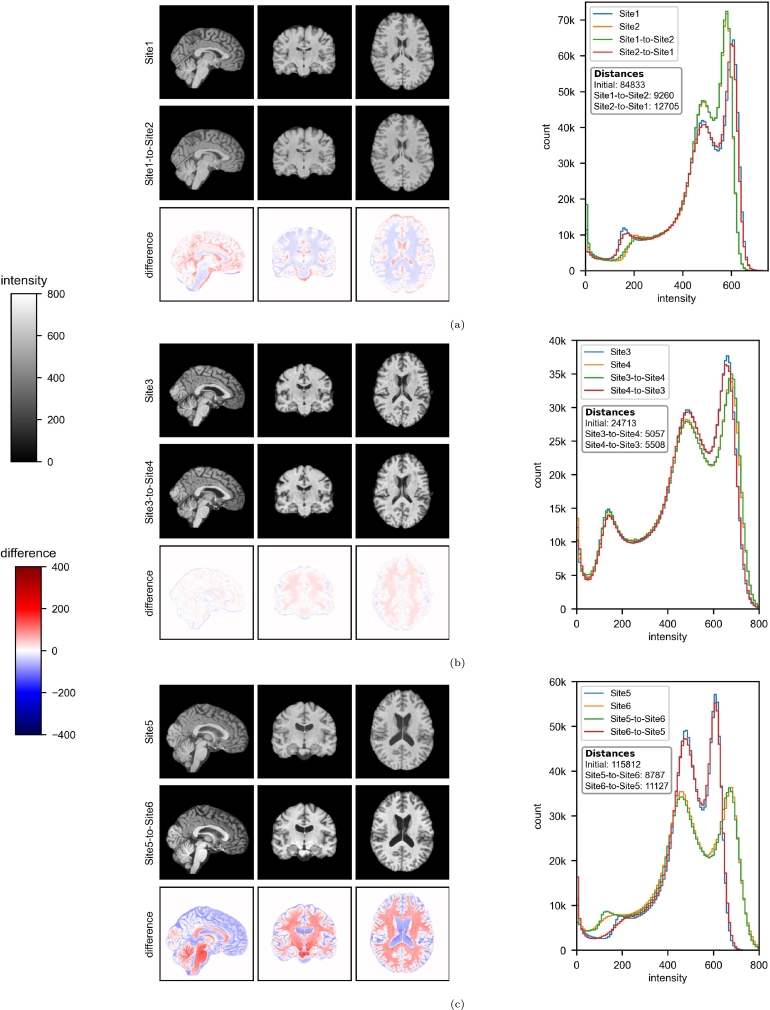
Slices and averaged histograms of brain intensities in the paired-site experiments. One MRI was randomly sampled for each illustrated harmonization. The differences correspond to a voxel-wise subtraction, i.e., the harmonized image minus the original image. 100 consecutive intensity bins are defined from 0 to 900 for the averaged histograms. Euclidean distances in the absence of harmonization and Euclidean distances between harmonized images and corresponding target histograms are shown. (a) Site1/Site2; (b) Site3/Site4; (c) Site5/Site6.

Each pair of sites showed more or less variabilities on the distribution of voxel intensities (Fig. 2). The difference in contrast was significant between Site5 and Site6, but it was well corrected by harmonization (Fig. 2c). The differences were more subtle for Site1/Site2 and Site3/Site4, but harmonization still managed to homogenize the distributions (Fig. 2a and 2b). The six harmonization procedures decreased Euclidean distance [69] between averaged histograms of brain intensities by a mean of 85.69%.

#### 3.1.2 Site-related variabilities of MRI features

**Figure 3:**
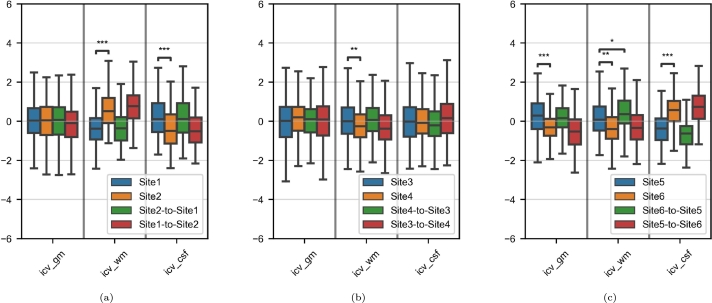
Boxplots of the tissue volumes in the paired-site experiments. The volumes are divided by the total intracranial volume. For each subfigure and each tissue, the y-axis is a Z-score based on the two sets of real images. Asterisks indicate significant t-tests before and after harmonization against the corresponding target sets (*: p < 0.05; **: p < 0.01; ***: p < 0.001). (a) Site1/Site2; (b) Site3/Site4; (c) Site5/Site6.

The brain volumes were significantly associated with the site for the Site1/Site2 and Site5/Site6 pairs (Fig. 3a and Fig. 3c, respectively). For the Site3/Site4 pair, only WM volumes showed significant site-related variability (Fig. 3b). After harmonization, the inter-site variabilities were lower and only the difference in WM volume between Site5 and Site6 was still statistically significant (p=0.0461).

**Figure 4:**
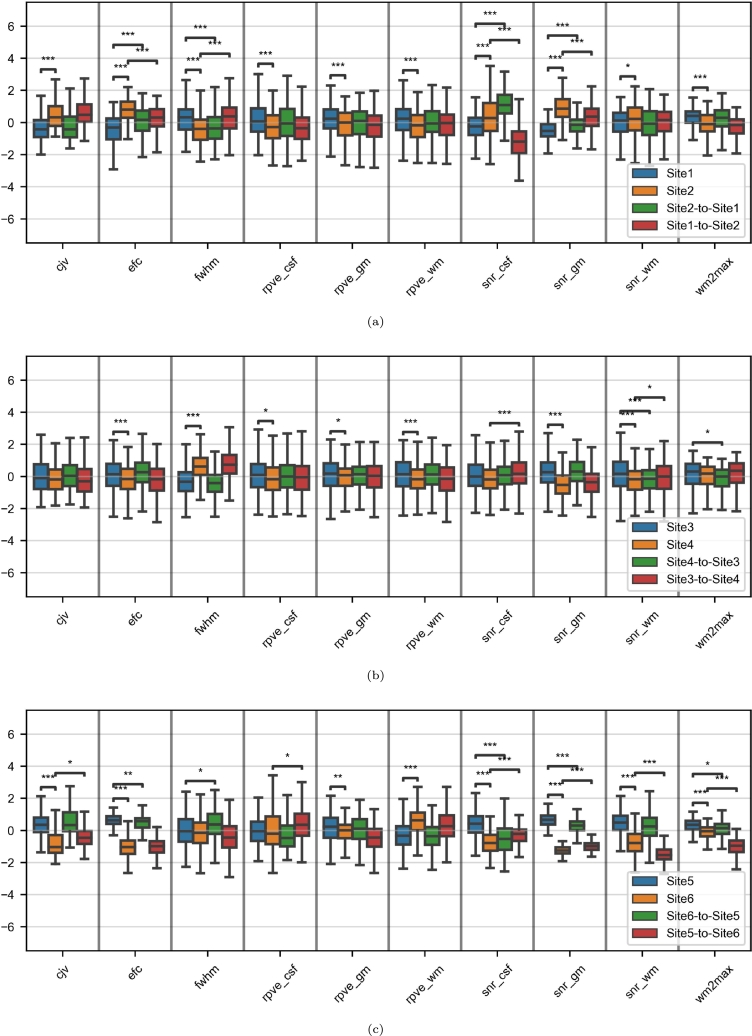
Boxplots of the image quality metrics in the paired-site experiments. For each subfigure and each metric, the y-axis is a Z-score based on the two sets of real images. Asterisks indicate significant t-tests before and after harmonization against the corresponding target sets (*: p < 0.05; **: p < 0.01; ***: p < 0.001). (a) Site1/Site2; (b) Site3/Site4; (c) Site5/Site6.

A majority of the IQMs presented site effects in the three experiments before harmonization (Fig. 4). These effects were particularly significant for the Site1/Site2 and the Site5/Site6 pairs (Fig. 4a and 4c, respectively). For most of them (e.g. *cjv* and partial volume effects), harmonization was effective in both directions - this was particularly true for the Site3/Site4 pair (Fig. 4b) - but not for *snr_csf*, where harmonization led to chaotic changes in most experiments.

**Figure 5:**
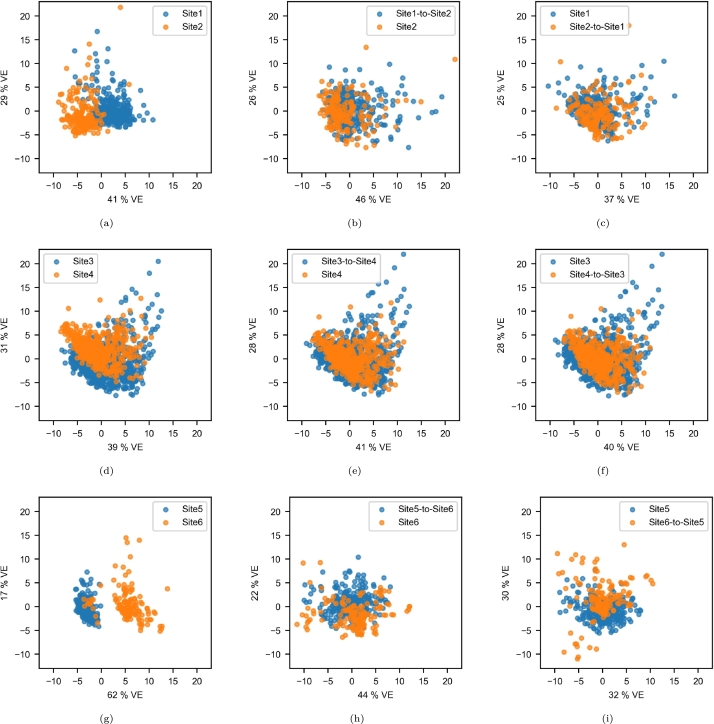
Components of the PCA of the radiomic features in the paired-site experiments. The x and y axes correspond to the first and second principal axes, respectively. VE: variance explained. (a) Site1/Site2; (b) Site1-to-Site2/Site2; (c) Site1/Site2-to-Site1; (d) Site3/Site4; (e) Site3-to-Site4/Site4; (f) Site3/Site4-to-Site3; (g) Site5/Site6; (h) Site5-to-Site6/Site6; (i) Site5/Site6-to-Site5.

In Fig. 5, we present the results of the PCA of radiomic features. The dissociation was clear for the Site5/Site6 pair (Fig. 5g) and after harmonization, the samples were more mixed even if site clusters were still distinguishable (Fig. 5h and 5i). The site effect was less clear but still significant for Site1 and Site2 (Fig. 5a) and was also successfully reduced after harmonization (Fig. 5b and 5c). Even if the Site3 and Site4 samples were less clustered (Fig. 5d), higher overlaps were obtained as well (Fig. 5e and 5f).

#### 3.1.3 Brain age prediction

**Figure 6:**
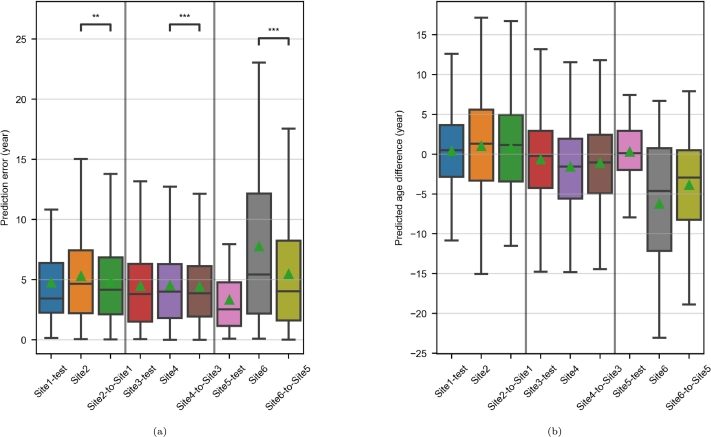
Age prediction in the paired-site experiments. (a) Prediction errors, asterisks indicate significant Wilcoxon signed-rank tests (*: p < 0.05; **: p < 0.01; ***: p < 0.001). (b) Predicted age differences, computed for each MR image as the predicted age minus the chronological age.

Fig. 6 illustrates the results related to the three age prediction experiments. In all three experiments, the MAE of the test dataset was lower than that of the generalization dataset, and harmonization resulted in a significant decrease of the prediction errors – even if the size of the decrease varied (Fig. 6a). For the three generalization sets, the MPAD was closer to 0 with harmonization (particularly for the Site5/Site6 pair), meaning that under/overestimation patterns caused by site effects were partly corrected on the harmonized MR images (Fig. 6b).

#### 3.1.4 Radiologic scores

**Figure 7:**
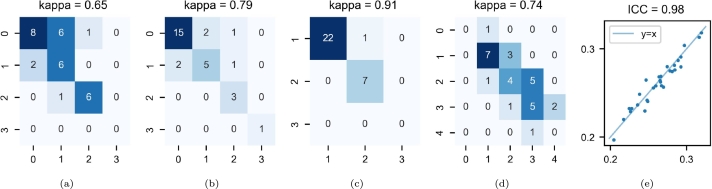
Radiologic scores in the paired-site experiments. The results are represented in a contingency table for ordinal variables (color depth is proportional to sample size for each outcome) and in a scatter plot for Evans index. The y- and x-axes correspond to the values of the original and harmonized MR images, respectively. (a) global cortical atrophy; (b) medial temporal atrophy; (c) number of enlarged perivascular spaces in the basal ganglia; (d) number of enlarged perivascular spaces in the centrum semiovale; (e) Evans index.

The consistency of the radiologic assessments before and after harmonization was good for GCA (Fig. 7a) and EPS-CS (Fig. 7d) and excellent for MTA (Fig. 7b), EPS-BG (Fig. 7c) and Evans index (Fig. 7e).

### 3.2 Multisite experiment

#### 3.2.1 Image and histogram comparisons

**Figure 8:**
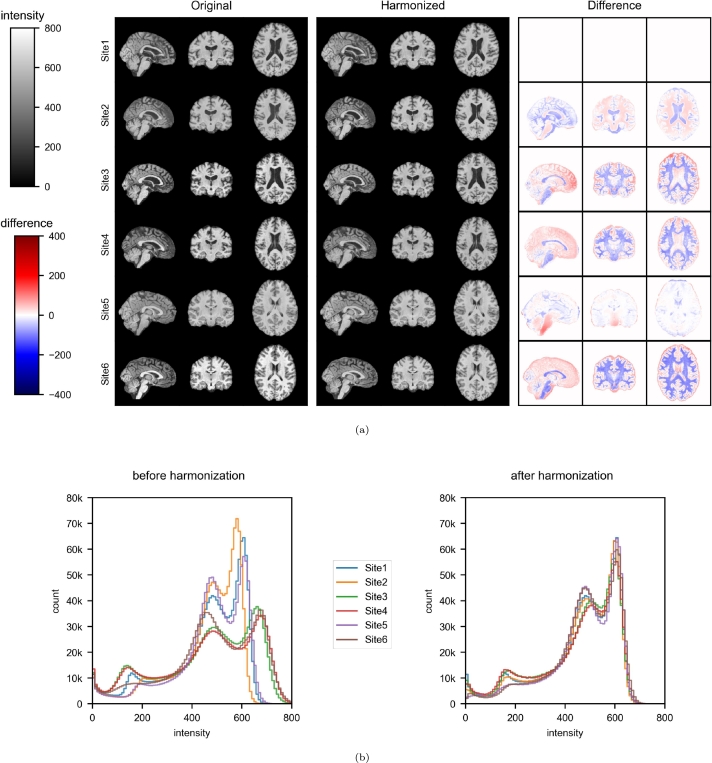
Slices and averaged histograms of brain intensities in the multisite experiment. (a) One MR image was randomly sampled from each site and slices are displayed before and after harmonization against Site1. The differences correspond to a voxel-wise subtraction, i.e. the harmonized image minus the original. (b) Averaged histogram of brain intensities is computed for each site with 100 consecutive intensity bins from 0 to 900.

Fig. 8a shows that the original Site3, Site4 and Site6 images were brighter and had a high GM/WM contrast, whereas the images at Site2 and Site5 were darker than those at Site1. These inter-site variabilities were no longer visible on the harmonized MR images. Nevertheless, the individual brain structures appeared to have been conserved.

In the absence of harmonization, the averaged brain intensity histograms differed from one site to another (8b). To quantify these differences, we computed the sum of the 100 standard deviations of the mean voxel count per site associated with the 100 histogram bins and obtained a value of 465197. The histograms were more similar after harmonization and our index of inter-site heterogeneity fell to 135383 (a 70.90% decrease).

#### 3.2.2 Site-related variabilities of MRI features

**Figure 9:**
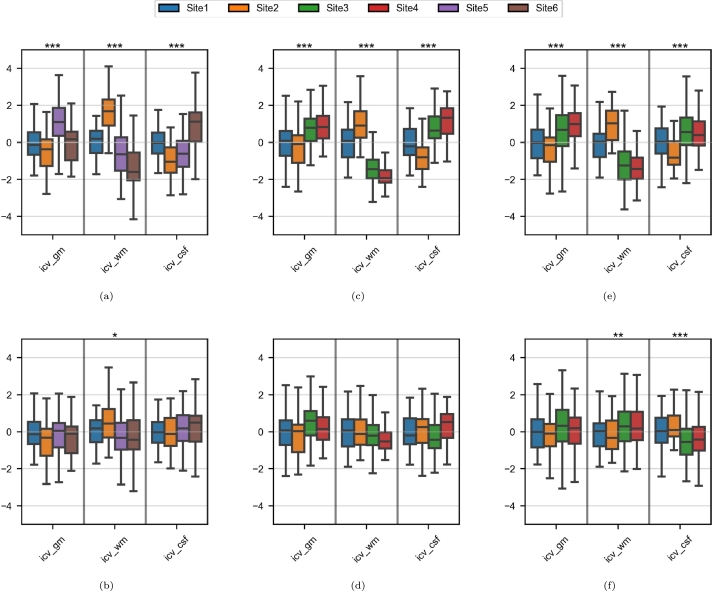
Boxplots of the tissue volumes in the multisite experiment. The volumes are divided by the total intracranial volume. For each subfigure and each tissue, the y-axis is a Z-score based on the Site1 samples in the given age range. Asterisks indicate significant ANOVA tests (*: p < 0.05; **: p < 0.01; ***: p < 0.001). (a) 20-30 age range before harmonization; (b) 20-30 age range after harmonization; (c) 50-60 age range before harmonization; (d) 50-60 age range after harmonization; (e) 60-70 age range before harmonization; (f) 60-70 age range after harmonization;

The measure for each tissue volume in each age range varied significantly from one dataset to another (Fig. 9a, 9c, 9e). The variability was lower after harmonization (Fig. 9b, 9d, 9f), except for the CSF volumes in the 60-70 age range (Fig. 9f).

**Figure 10:**
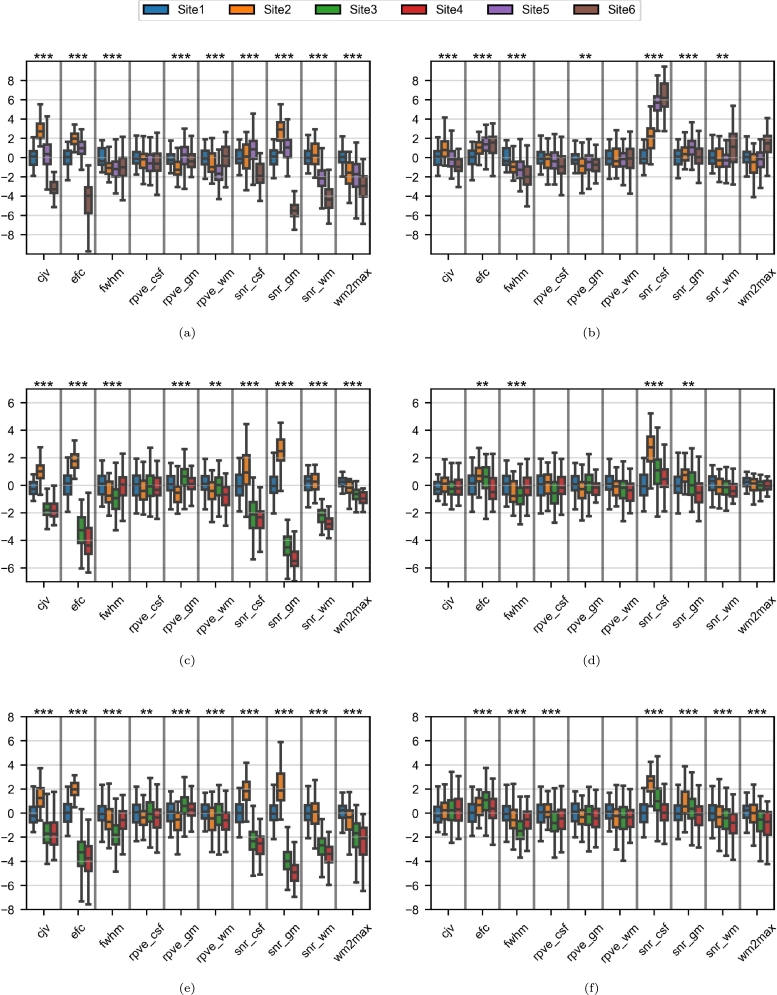
Boxplots of the image quality metrics in the multisite experiment. For each subfigure and each tissue, the y-axis is a Z-score based on the Site1 samples in the given age range. Asterisks indicate significant ANOVA tests (*: p < 0.05; **: p < 0.01; ***: p < 0.001). (a) 20-30 age range before harmonization; (b) 20-30 age range after harmonization; (c) 50-60 age range before harmonization; (d) 50-60 age range after harmonization; (e) 60-70 age range before harmonization; (f) 60-70 age range after harmonization;

Large inter-site differences in almost all the IQMs were apparent on the original MR images (Fig. 10a, 10c, 10e). Overall, the differences were reduced by harmonization (Fig. 10b, 10d, 10f) except for *fwhm* and *snr_csf*.

**Figure 11:**
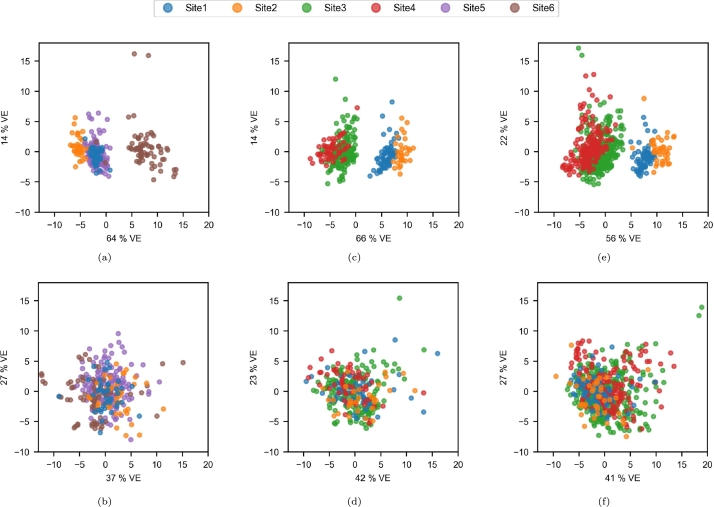
Components of the PCA of the radiomic features in the multisite experiment. The x and y axes correspond to the first and second principal axes, respectively. VE: variance explained. (a) 20-30 age range before harmonization; (b) 20-30 age range after harmonization; (c) 50-60 age range before harmonization; (d) 50-60 age range after harmonization; (e) 60-70 age range before harmonization; (f) 60-70 age range after harmonization;

Based on the PCA components extracted from the radiomic features, site clusters were easily distinguished for the original data (Fig. 11a, 11c, 11e). An exception was seen for the 20-30 age range at Site1, the components of which were superposed on those of Site5 (Fig. 11a). The clusters were not more easily distinguished after harmonization (Fig. 11b, 11d, 11f).

#### 3.2.3 Brain age prediction

##### 3.2.3.1 Single-site training set

**Figure 12:**
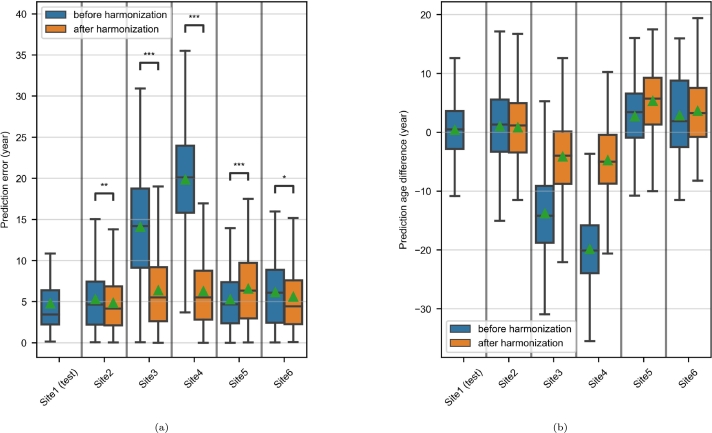
Age prediction by the model trained on Site1 in the multisite experiment. (a) Prediction errors, asterisks indicate significant Wilcoxon signed-rank tests (*: p < 0.05; **: p < 0.01; ***: p < 0.001). (b) Predicted age differences, computed for each MR image as the predicted age minus the chronological age.

Fig. 12 illustrates the results produced by the brain age prediction model trained on Site1 and applied to all our MRI datasets. Fig. 12a shows that, as in the paired-site experiments, the test set's MAE was lower than those of the generalization sets. After harmonization, the prediction errors fell significantly for Site2, Site3, Site4 and Site6. In contrast, they were significantly higher for Site5. It can be seen in Fig. 12b that, after harmonization, the MPAD was closer to zero for Site2, Site3 and Site4 but further from zero for Site5 and Site6; this finding suggests that site-related under/overestimation patterns were attenuated in the first case and accentuated in the second. For each set of predictions (other than for Site1), the MPAD had the same sign as the TMD, which reflects RTTM.

##### 3.2.3.2 Multisite training set

We set up two brain age prediction experiments to evaluate the effect of harmonization on a large, multicenter training set. The harmonization procedure reduced the MAE from 4.48 before harmonization to 3.91 afterwards. The errors were significantly lower after harmonization (p=0.0033). Further results are provided in the Supplementary Materials (section 8).

#### 3.2.4 Correlation between gray-matter volume and age

**Figure 13:**
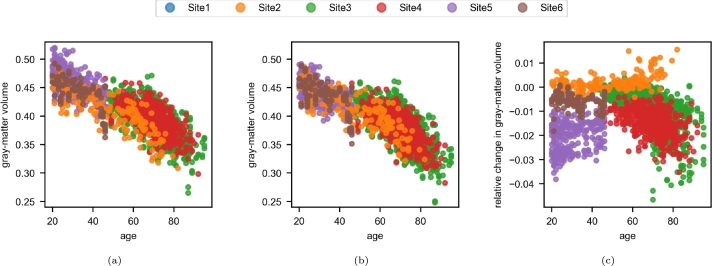
Plots of gray-matter volume by age in the multisite experiment. The volumes are divided by the total intracranial volume. (a) before harmonization; (b) after harmonization; (c) relative change, computed for each MR image as the relative volume after harmonization minus the one before harmonization.

Harmonization slightly reduced the dispersion around the linear central trend for GM volumes with age (Fig. 13a and 13b). The strength of the negative linear correlation changed significantly from -0.816 before harmonization to -0.821 after harmonization (p=0.0014 in Steiger's test). The volume estimations tended to be lower after harmonization except for Site2 and for Site3 and Site4, the decrease tended to be more important with age (Fig. 13c).

#### 3.2.5 Radiologic scores

**Figure 14:**
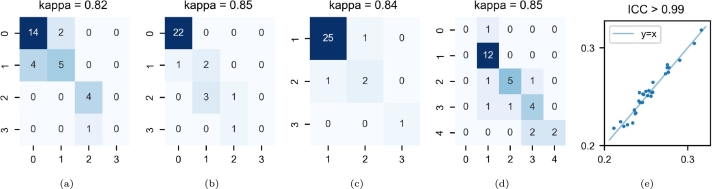
Radiologic scores in the multisite experiment. The results are represented in a contingency table for ordinal variables (color depth is proportional to sample size for each outcome) and in a scatter plot for Evans index. The y- and x-axes correspond to the values of the original and harmonized MR images, respectively. (a) global cortical atrophy; (b) medial temporal atrophy; (c) number of enlarged perivascular spaces in the basal ganglia; (d) number of enlarged perivascular spaces in the centrum semiovale; (e) Evans index.

The consistency of the radiologic assessments before and after harmonization was excellent for GCA (Fig. 14a), MTA (Fig. 14b), EPS-BG (Fig. 14c), EPS-CS (Fig. 14d) and Evans index (Fig. 14e).

### 3.3 Harmonization on traveling subjects

Table 3 reports the SSIMs obtained with the traveling-subject dataset. Our 3D model resulted in a significant increase in SSIMs when harmonizing from Site3 to Site4 (p < 0.001) and a significant decrease when harmonizing from Site4 to Site3 (p < 0.001). In both cases, the 2D CycleGAN significantly reduced the SSIMs (p < 0.001). However, the SSIMs obtained with our 3D model were significantly higher in both harmonization directions (p < 0.001).

**Table 3:** SSIM in the traveling-subject dataset.

	No harmonization	Site3 → Site4		Site4 → Site3	
		2D CycleGAN	3D CycleGAN	2D CycleGAN	3D CycleGAN
SSIM ⁎	0.9523 ± 0.0131	0.9407 ± 0.0111	0.9533 ± 0.0126	0.9454 ± 0.0105	0.9499 ± 0.0131

^⁎^ SSIM is expressed as mean ± standard deviation.

## 4 Discussion

In the present study, we developed a 3D domain transfer model for the inter-site harmonization of T1w brain images. In view of the known concerns about the technical requirements of deep learning model and their reliability with miscellaneous MR data [70], we were looking for the efficient use of computation resources and robustness to various datasets. Based on a pool of MR images acquired with six different machines, our experiments showed that the model could reduce inter-site variabilities in intensity distributions, brain volumetry, quality metrics and radiomic features. Harmonization was also associated with significantly more accurate brain age prediction (whether with small single-site or large multicenter training sets) and mitigated site effects on changes in GM volume with age. The radiologic scores also confirmed the consistency of biological information at the individual level before and after harmonization. Additionally, validation with a traveling-subject dataset indicated a superiority over a well-known 2D domain transfer model.

In contrast to earlier research on models that processed parts of images only (slices or 3D patches), our model was designed to operate on whole 3D T1w brain images. We compensated for the additional computing costs by using a Unet architecture for the generators, which is less greedy than the standard Resnet architectures used in CycleGAN approaches [17], [25], [18], [19]. We opted for transposed convolutions because resize convolutions [71], [19] lost more of the anatomic information in the input MR images (data not shown) and produced more cumbersome networks. Dewey et al. [11] also found that transposed convolution was more suitable for harmonization. However, contrary to Dewey et al., our generator architecture consisted only of strided convolutions. This choice helped to reduce computational requirements but could be the source of some remaining inter-site heterogeneities in the IQMs after harmonization (sections 3.1.2 and 3.2.2), as transposed convolutions are susceptible to artifacts [11], [71].

Our model's CycleGAN structure enabled it to tackle the problem of biological differences between the site populations, as shown by the biased sampling strategy set up to avoid the correction of age effects in the multisite experiment (section 2.4.1). The experiments without a biased sampling strategy led to significant losses in age-related variabilities (data not shown), which is in line with a previous report on brain tumors [72]. When compared with the removal of MR images outside a specific age range [13], our sampling strategy better conserved the diversity in the training data. Style transfer methods have been used to tackle biological differences between sites, by ignoring site information [21] or by introducing biological conservation modules [23]. However, in contrast to our experiments on age variability (sections 3.2.3 and 3.2.4), the above-cited studies did not assess the conservation or accentuation patterns related to imbalance variables.

The results of our paired-site experiments (section 3.1) generally confirmed the model's efficiency with various source and target datasets with differences in sample size, age distribution, scanner used and acquisition parameters (section 2.1.1). The data at Site5 and Site6 came from different studies (i.e. NKI-RS and NMorphCH) and differed markedly. The data at Site1 and Site2 came from the same database (IXI) and were more homogeneous. However, our results suggested the presence of clear site-related variabilities — probably because of the difference in field strength (i.e. 1.5 vs. 3 Tesla). The differences between the Site3 and Site4 datasets (both from the OASIS-3 study) were smaller but our model still managed to correct them significantly. This is a strong point, given that some harmonization methods harmed datasets with small or no site effects [73].

The multisite experiment enabled us to evaluate the model in a more common situation, i.e. data from more than two sites, and harmonization into a common space. Although some site-related differences were still present after harmonization (mainly among the participants in the 60-70 age range), our analysis of the intensity distributions (section 3.2.1) and the MRI features (section 3.2.2) indicated that the datasets had indeed been uniformized. Harmonization significantly improved the performance of the brain age prediction model trained on Site1's MR images for all sites except Site5 (section 3.2.3.1). When considering the predicted age differences and the TMD, RTTM appeared to be greater after harmonization of Site5 MR images. We speculate that site-related variabilities led to age underestimation in these images, and this fortuitously compensated for RTTM, resulting in age overestimation in younger participants. To validate this hypothesis, we included older participants in the dataset (section 9 of the Supplementary Materials).

Thus, the age prediction results highlighted the advantages of our method for domain adaptation, i.e. when a predictive model is trained with data from one site and applied to others (sections 3.1.3 and 3.2.3.1). In a similar manner, Bashyam et al. [27] achieved significant improvements in predicting age with harmonization. We further demonstrated the predictive value of our model with a large multicenter training set (section 3.2.3.2); this was not necessarily expected because the quantity and diversity of the data might have favored the distinction between site- and age-related patterns. Robinson et al. [26] performed a similar experiment but with only two sites and did not report on the extent of the improvements.

Our analysis of the correlation between GM volume and age (section 3.2.4) provided a view of a specific brain aging pattern. The relation was significantly more linear after harmonization. Similarly, Fortin et al. [7] validated the harmonization of cortical thickness across sites and scanners but did not process the entire MR image. Furthermore, the initial linear correlation observed by Fortin et al. (-0.70) was weaker than that observed in the present study (-0.82) and so was easier to reinforce.

The radiologic scores (sections 3.1.4 and 3.2.5) showed that our model was able to conserve precise radiologic information related to brain atrophy, perivascular spaces, and ventricle size. In some previous studies of medical image translations, generated images were radiologically assessed for realism [7], [74], [75]. In the present study, however, we wanted to investigate the conservation of individual features with harmonization. Indeed, it is known that the application of CycleGAN approaches may result in the loss of valuable information from the input images [72].

Our validation on the traveling-subject dataset (section 3.3) shows that our 3D CycleGAN model is more effective than the classical 2D CycleGAN in harmonizing Site3 and Site4 MR images. With our harmonization from Site3 to Site4, we achieved a significant increase in the SSIM compared to MR images after preprocessing. Conversely, SSIM decreased when harmonizing from Site4 to Site3. This can be partially explained by the limited inter-site variability between these two sets, as indicated by the results of the corresponding paired-site experiment (section 3.1). However, this mitigated outcome of harmonization from Site4 to Site3 is counterbalanced by the substantial improvement it enabled in the brain age prediction of the model trained on Site3 MR images and applied to Site4 MR images (section 3.1.3).

The present study has some limitations. Firstly, the CycleGAN approach requires an independent training for each site. Nonetheless, the great diversity of input datasets and the large number of evaluations performed in the present study (relative to the typical literature findings) show the interest of our model for datasets comprising a relatively large number of MR images; Gebre et al. [20] also showed the value of CycleGAN against ComBat approaches, conditional GAN and style transfer methods for the harmonization of large cross-sectional datasets. Secondly, we opt for a comparison between our 3D method and 2D CycleGAN due to its established status and the availability of implementation details [16]. However, we acknowledge that other recent methods [23], [22] could also be evaluated. Nevertheless, performing a fair comparison would pose a significant challenge due to various factors, including the difficulty in accessing and using the implementations, as highlighted by Hu et al. [5], the heterogeneity of the data used for development, and differences in the preprocessing (e.g. skull-stripping and rescaling). Thirdly, many of the features that we used to analyze site effects are dependent on the segmentation method (FSL-FAST here), and so our results might have been different if another method had been applied. However, our use of a variety of measures provided valuable information and limited the impact of the segmentation tool. Fourthly, despite the generic nature of our method, we only studied the harmonization of T1w brain images. Although T1w sequences are used in many research studies and in clinical practice (e.g. in dementia and multiple sclerosis), the use of multimodal approaches might improve harmonization [11]. Lastly, our model was only applied to data from apparently healthy participants. We now intend to test it on data from people with diagnosed neurologic disorders, notably so that we can analyze changes in markers (e.g. lesions) with disease progression. We also plan to extend our CycleGAN framework to combine its harmonization capacity with the convenience of methods that can be applied to any image after training.

## 5 Conclusions

In this article, we propose an unsupervised model for the inter-site harmonization of T1w MR images of the brain. This optimized 3D deep learning approach processes whole brain images and is robust to diverse MRI datasets. A range of experiences on various cohorts at different scales attests to the model's ability to eliminate diverse inter-site variabilities, conserve radiologic information and reinforce biological patterns. Despite the presence of major biological differences between sites, our choice of an appropriate training strategy helped to make the multisite harmonization a success. Our extensive validation of the harmonization results is promising for various future applications in multicenter studies.

## CRediT authorship contribution statement

**Vincent Roca:** Conceptualization, Formal analysis, Investigation, Methodology, Software, Validation, Visualization, Writing – original draft, Writing – review & editing. **Grégory Kuchcinski:** Conceptualization, Investigation, Supervision, Writing – review & editing. **Jean-Pierre Pruvo:** Resources. **Dorian Manouvriez:** Resources. **Xavier Leclerc:** Resources, Writing – review & editing. **Renaud Lopes:** Conceptualization, Funding acquisition, Methodology, Project administration, Resources, Supervision, Writing – review & editing.

## Declaration of Competing Interest

The authors declare the following financial interests/personal relationships which may be considered as potential competing interests: Vincent Roca reports financial support was provided by Philips France Commercial.

## Data Availability

All the MR images used in this study come from public databases (section 2.1.1). The selection of participants is detailed in the Supplementary Materials (section 1). The features extracted to generate the results will be made available on request. The Python code for the harmonization model, the age prediction model, the IQM extraction, the computation of tissue volumes and the function used for balancing the age distributions is available in an online repository: https://gitlab.com/RocaV/3d_cyclegan_mri_harmonization.

## Appendix A Supplementary material

MMC Supplementary materials about methods and results. MMC

## Footnotes

1 https://brain-development.org/ixi-dataset/ Accessed: 2022-01-15.
