# Supplementary material for: A three-dimensional deep learning model for inter-site harmonization of structural MR images of the brain: Extensive validation with a multicenter dataset
Source: Heliyon. 2023 Nov 23;9(12):e22647. doi: 10.1016/j.heliyon.2023.e22647 (PMC10724680; doi:10.1016/j.heliyon.2023.e22647)
Supplement: MMC — Supplementary materials about methods and results. [file mmc1.pdf]

Supplementary Materials

1 MRI data selection

1.1 IXI

We only used *HH* and *Guys* images. Participants with missing data for age or with several contradictory indications of age in the metadata were excluded. Four participants from *HH* and four from *Guys* were excluded because the brain was too large along the anteroposterior axis in MNI space ($>$ 192 voxels).

1.2 OASIS-3

We only used T1w brain images acquired with a *Biograph_mMR* or *TrioTim* scanner and where the CDR was 0. Images with missing metadata for age were excluded. Next, to avoid overlap, all participants present in both groups were removed from the *TrioTim* group. One MR image was excluded because of poor quality (no brain on the image). Seven MR images from *Biograph_mMR* and one from *TrioTim* were excluded because the brain was too large along the anteroposterior axis in MNI space ($>$ 192 voxels).

1.3 NMorphCH

All the images were included in the present study.

1.4 NKI-RS

Given the need for a dataset of young participants (i.e. with an age distribution similar to that of NMorpCH), only MR images of participants aged between 20 and 46 were included in the study. We found that the intensity distribution of MR images from the *NFB3* session was very different from that of other images, and so we decided to remove them from the dataset. Three MR images were excluded because the brain was too large along the anteroposterior axis in MNI space ($>$ 192 voxels).

2 Details of CycleGAN training

We adopted a least squares loss function for the adversarial training [3] as we found it to be more adapted than the Wasserstein loss [1] for mixed precision computations. Each training of an harmonization model consisted of 300 epochs, each of which comprised 200 steps. We used an Adam optimizer [2] with a learning rate initialized to 0.0002 for the first 150 epochs and linearly decayed to zero over the next 150 epochs. The training took around 20 hours to run on an NVIDIA Quadro RTX 6000 GPU with TensorFlow v2.9.1. A data augmentation consisted of a random translation ($\pm$ 5 voxels) in the three orthogonal planes.

The code is available in our online repository.

3 Comparison of the age distributions in the paired-site experiments

Using a p-value threshold of 0.05, none of the three dataset pairs had age distributions significantly different (Table S1, the ages were averaged by subject and the p-values were corrected with the Benjamini-Hochberg procedure).

4 Age distribution per dataset

Fig. S1 presents the probability distribution of age for each dataset.

Table S1: Mann-Whitney U test between the age distributions in the paired-site experiments.

**dataset pair**	Site1/Site2	Site3/Site4	Site5/Site6
**p-value**	0.0678	0.0678	0.2648

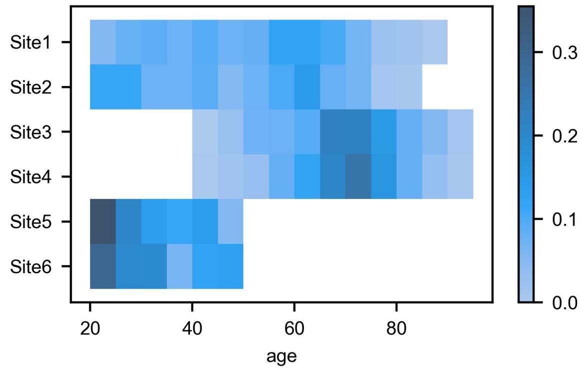

Figure S1: The age distribution in each dataset. Displays the proportion of MR images in each age range for each dataset.

5 Sampling strategy for the balancing of age distributions

5.1 Procedure

To homogenize the age distributions in two datasets A and B, we used a function that takes the list of ages in A, the list of ages in B, and a set of consecutive age ranges as inputs and then outputs a sampling probability distribution (i.e. a probability associated with each input age range).

First, we computed the number of MR images within each age range for A and for B, together with the corresponding probability distributions. The algorithm then modified the two distributions iteratively. In each step, the age range with the smallest number of MR images (considering A and B separately) not yet fixed was selected and fixed; the probabilities of the dataset with the largest number of MR images in this age range were then modified by setting the corresponding probability to the value in the other dataset and by updating the other probabilities not yet fixed. After a number of steps equal to the number of bins minus 1, the two probability distributions were equal and corresponded to the sampling distribution.

The code is available in our online repository.

5.2 Sampling probabilities in the multisite experiment

Fig. S2 shows the probability distributions for age and for sampling in each age-imbalanced dataset pair in the multisite experiment.

6 Training details of brain age prediction

All the results presented here are based on age prediction models trained with 400 epochs. The data augmentation consisted in a random shift (± 5 voxels) in the three orthogonal planes and a random rotation (± 10 °) in one randomly selected plane. The random rotation was applied half the time. We did not use a validation set because it was not of value with our dataset sizes. As in the harmonization procedure, we adopted a mixed precision policy. The batch size was set to 16. We used an Adam optimizer with a linear learning rate decay from 0.001 to 0.0001.

The code is available in the online repository.

7 2D CycleGAN

We reproduced the network architectures and the training procedure described by [4] except for the number of input/output channels (1 instead of 3) and the final activation of the generator which we replaced by the same we used for our 3D CycleGAN. For each inference, we applied the original brain mask to the generated volume to avoid background artifacts. The number of training steps per epoch was fixed to 5000.

8 Age prediction errors per site with the multicite training set

Fig. S3 shows the age prediction errors per site for the models trained on a multicenter dataset in the multisite experiment.

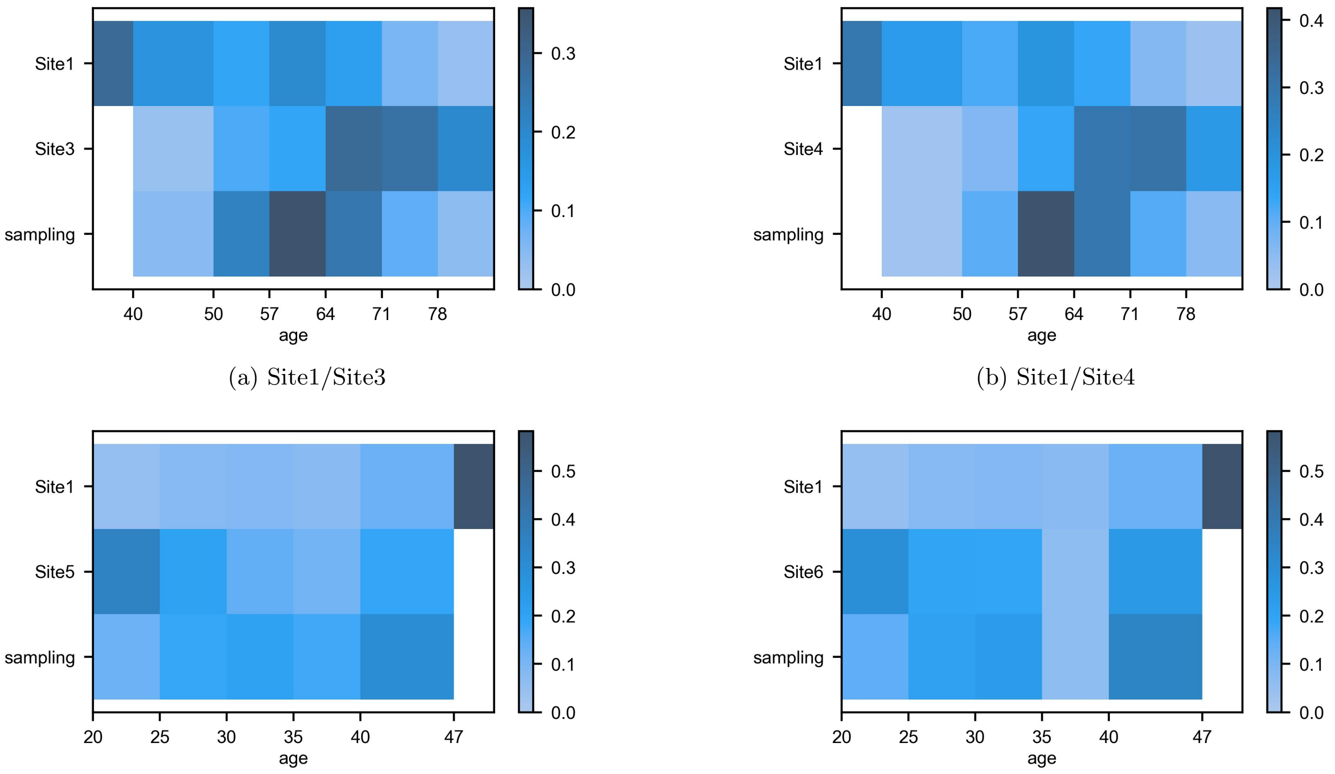

(a) Site1/Site3

(b) Site1/Site4

(c) Site1/Site5

(d) Site1/Site6

Figure S2: Probability distributions for age and for sampling in each age-imbalanced pair of datasets in the multisite experiment. The graduations on the x-axis indicate the age ranges used for bias sampling. The color intensities indicate the proportions for each range.

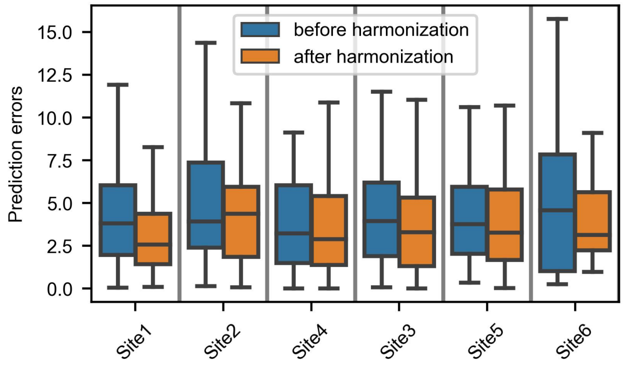

Figure S3: Boxplots of the age prediction errors per site for the models trained on a multicenter dataset in the multisite experiment.

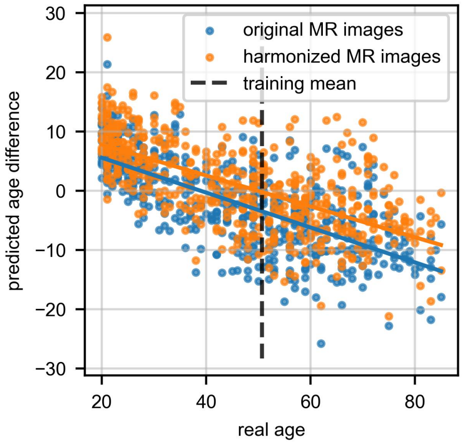

Figure S4: Predicted age difference as a function of real age in the extended Site5 dataset. The predicted age difference is computed as the predicted age minus the real age. For each MRI set, a linear least-squares regression line is plotted.

9 Additional age prediction results on Site5

We applied the age prediction model trained with Site1 to an extended version of Site5; it included older participants that were not included in the main study (see 1.4). We harmonized all the Site5 MR images against Site1 with the Site1/Site5 model previously trained in the multisite experiment (i.e. with young participants only, see section Fig. S2c). The MAE was 6.54 years (MPAD: 2.07) on the original MR images and 6.12 (MPAD: 1.02) on the harmonized MR images.

Fig. S4 shows the predicted age differences, as a function of the real age. In the absence of harmonization, the intersection between the training mean line and the regression line is clearly below zero on the y-axis, which suggests that age was indeed underestimated. After harmonization, the intersection is close to zero — suggesting that the underestimation is no longer present.

References

[1] Ishaan Gulrajani et al. "Improved training of wasserstein gans". In: *Advances in neural information processing systems* 30 (2017).

[2] Diederik P. Kingma and Jimmy Ba. *Adam: A Method for Stochastic Optimization*. 2017. DOI: 10.48550/ARXIV.1412.6980.

[3] Xudong Mao et al. "Least Squares Generative Adversarial Networks". In: *2017 IEEE International Conference on Computer Vision (ICCV)*. IEEE, Oct. 2017. DOI: 10.1109/iccv.2017.304.

[4] Jun-Yan Zhu et al. "Unpaired Image-to-Image Translation Using Cycle-Consistent Adversarial Networks". In: *2017 IEEE International Conference on Computer Vision (ICCV)*. IEEE, Oct. 2017. DOI: 10.1109/iccv.2017.244.
